# Effects of different support surfaces on postural stability and sensorimotor cortex activation among individuals with chronic ankle instability

**DOI:** 10.3389/fspor.2026.1754699

**Published:** 2026-03-09

**Authors:** Yurui Shen, Mengyu Liu, Ting Lai, Jingkun Zhang, Chen Yang, Liangliang Zheng

**Affiliations:** 1 College of Sports and Health, Shandong Sport University, Jinan, China; 2 College of Sports and Health, Nanjing Institute, Nanjing, China

**Keywords:** ankle sprains, cerebral cortex activation, postural control, single-leg stance, unstable surface

## Abstract

**Objectives:**

Chronic ankle instability (CAI) is characterized by recurrent ankle sprains and impaired postural control, which are particularly evident on unstable surfaces. Evidence indicates that the central nervous system undergoes adaptive plastic changes; however, the specific cortical mechanisms involved remain unclear. Therefore, this study aimed to investigate postural stability and cortical activation in individuals with CAI, especially on unstable surfaces, in order to identify compensatory cortical strategies underlying impaired postural control.

**Methods:**

Seventeen people with CAI and seventeen without CAI were recruited. Static postural control was measured under two experimental conditions: a stable surface and an unstable surface simulated by a foam pad. Static postural control was measured during single-leg standing and was represented by the root mean square (RMS) of the plantar center of pressure (COP). Cerebral cortex oxyhemoglobin concentration (ΔHbO₂) was measured using functional near-infrared spectroscopy (fNIRS). Two-way mixed ANOVA (between group: CAI vs. non-CAI, within group: stable vs. unstable surface) was used to analyze data.

**Results:**

Across different support surfaces, distinct patterns of between-group differences were observed. Under the stable surface condition, CAI group exhibited significantly lower sample entropy in the mediolateral direction of the center of pressure (SampEn_ML) compared with the non-CAI group. In contrast, under the unstable surface condition, the between-group differences were primarily reflected in increased COP sway magnitude and cortical activation. Moreover, during single-leg standing on the unstable surface, the CAI group demonstrated a significantly larger 95% confidence ellipse area (95%Area), greater root mean square of anteroposterior COP displacement (COP_RMS_AP), and higher ΔHbO₂ levels in the primary somatosensory cortex (S1), somatosensory association cortex (SAC), and the premotor and supplementary motor area (PMC & SMA) than the non-CAI group.

**Conclusions:**

Individuals with CAI exhibit impaired postural stability, particularly on unstable surfaces, accompanied by increased activation in the primary motor cortex, primary sensory cortex, somatosensory association cortex, and the premotor and supplementary motor area. These findings suggest a compensatory neural strategy, highlighting the critical role of cortical mechanisms in maintaining postural control in this population.

## 1 Introduction

Ankle sprains are among the most common musculoskeletal injuries in daily activities, and the risk of recurrent injury increases sixfold following an initial sprain (1). Approximately 20%–75% of affected individuals experience persistent symptoms and subsequently develop chronic ankle instability (CAI) (2). CAI is a sensorimotor dysfunction characterized by proprioceptive deficits, reduced postural stability, impaired neuromuscular control, and recurrent sprains (3, 4). It can also lead to early-onset osteoarthritis in up to 70% of patients (5). Additionally, ankle sprains account for an estimated $6.2 billion in medical costs among high school athletes in the United States (6).

Understanding the pathogenesis of CAI is essential for developing targeted rehabilitation strategies that enhance postural stability and reduce the risk of recurrent sprains. Prior research has shown that individuals with CAI exhibit diminished postural stability due to impaired peripheral receptors (7). This instability becomes more evident during single-leg stance, as indicated by shortened time to boundary (TTB) values and increased center of pressure (COP) excursions (8, 9). Moreover, compared with individuals without CAI, those with unilateral CAI require more time to achieve postural stability in the anteroposterior direction on both the affected and contralateral sides (10). However, peripheral mechanisms, such as damage to ligamentous mechanoreceptors, alone cannot fully account for these findings. Recent work suggests that sensorimotor deficits in CAI may arise not only from damaged ligamentous mechanoreceptors but also from adaptive changes within the central nervous system (11).

The sensorimotor cortex plays a central role in maintaining postural stability, and repeated ankle sprains in individuals with CAI may induced changes in cortical activation. Studies have reported increased activation in the primary sensory cortex (S1) during single-leg stance in individuals with CAI (12), as well as greater activation variability in the supplementary motor area (SMA) (5). However, Ma et al. found no significant differences in S1 activation levels between individuals with and without CAI (13), a discrepancy likely related to the stable support surface used in their protocol. Stable surfaces are comparatively less challenging and may fail to reveal subtle alterations in cortical activity. Conversely, tasks performed on unstable surfaces with multidirectional perturbations heighten postural demands (14), and offer deeper insights into how central regulatory mechanisms contribute to postural control in individuals with CAI.

Accordingly, this study aims to examine differences in postural control and sensorimotor cortex activation between individuals with and without CAI during single-leg stance on surfaces of varying stability. The findings are expected to support the development of assessment systems and rehabilitation programs that incorporate cortical activation patterns and instability-based evaluations. The hypotheses of this study are as follows: (1) In both groups, postural stability during single-leg stance is lower on an unstable surface than on a stable surface, and an unstable surface requires greater sensorimotor cortical activation. And (2) compared with individuals without CAI, those with CAI are expected to demonstrate a greater reduction in postural stability and a greater increase in sensorimotor cortical activation when standing on an unstable surface.

## 2 Materials and methods

### 2.1 Participants

An *a priori* power analysis (G*Power 3.1) was used to determine the sample size. To the best of our knowledge, no previous study has investigated the effects of different support surfaces on sensorimotor cortical activation during single-leg stance in individuals with or without CAI. Although some studies have examined the effects of different support surfaces on the center of pressure under the foot, they did not report effect sizes that could be used for sample size estimation. Therefore, a medium effect size (partial *η*2 p = 0.06), an alpha level of 0.05, and a statistical power of 0.90 were adopted in the present study, indicating that a total of 30 participants was required (15). To account for potential sample loss, 17 CAI participants and 17 non-CAI participants were recruited for this study (Table 1).

**Table 1:** Participant information (mean ± SD).

Index	CAI group	Non-CAI group	*T*-test	
	(*n* = 17)	(*n* = 17)	T value	*P* value
Age (years)	20.1 ± 1.5	20.9 ± 1.9	1.396	0.172
Height (cm)	173.4 ± 7.8	172.8 ± 8.5	0.209	0.835
Weight (kg)	67.9 ± 16.9	67.5 ± 12.4	0.081	0.936
Gender (male/female, *n*)	10/7	10/7	–	–
CAIT score (points)	16.4 ± 4.4	28.1 ± 1.2	−11.647	<0.001
Dominant side/non-dominant side	12/5	12/5	–	–
History of ankle injury (yes/no)	Yes	NO		

CAIT, Cumberland Ankle Instability Tool; CAI group, chronic ankle instability group; Non-CAI group, non-chronic ankle instability group.

The inclusion criteria for participants with CAI followed the guidelines of the International Ankle Consortium and the experimental design (16), which included: (1) age between 18 and 25 years; (2) a history of at least one severe ankle sprain accompanied by pain, swelling, and other inflammatory symptoms that resulted in an inability to participate in normal daily activities for more than one day; (3) the most recent ankle sprain occurring at least six months prior; (4) at least two episodes of ankle “giving way” within the past six months; and (5) a Cumberland Ankle Instability Tool (CAIT) score of less than 24 or a positive anterior drawer test. For people without CAI, the criteria required matching individuals with CAI in gender, age (±3 years), height (±5 cm), and weight (±5 kg), having a CAIT score of 24 or higher (17), and the absence of any history of ankle sprain or instability.

The exclusion criteria for both groups were (16): (1) a history of lower limb fracture or surgery; (2) an acute lower limb injury, such as a sprain, within the past three months; (3) the presence of neurological conditions that significantly impair motor function, including epilepsy; (4) bilateral CAI; and (5) Pes planus (flat feet). This study was approved by the Ethics Committee of Shandong Sport University (No. 2025046). All participants provided written informed consent form before the experiment.

### 2.2 Procedures

A three-dimensional force platform (AMTI, Inc. Watertown, MA, United States) was used to collect the COP displacement data during the balance task at a sampling rate of 1,000 Hz. To simulate an unstable support surface during the task, the medium-density memory foam pad (density = 108 kg/m³, 50 cm × 40 cm × 5 cm) was used to simulate an unstable support surface (14). This setup is widely used for the analysis of center of pressure and balance-related variables (18, 19). While COP data were being recorded using the force platform, a portable near-infrared imaging system (LIGHTNIRS, Shimadzu Corp, Kyoto, Japan) was used to measure the hemodynamic signals of the sensorimotor cortex during the single-leg stance task. The wavelengths were 780, 805, and 830 nm, with a sampling frequency of 13.3 Hz (20). Participants carried fNIRS while performing balance tasks and wore a whole-head fiber holder with standard head landmarks determined according to the international 10–20 system for EEG (21). The Cz electrode was located at the intersection of the line between the preauricular points and the line from the nasion to the inion. This configuration primarily targeted the primary motor cortex (M1), primary sensory cortex (S1), somatosensory association cortex (SAC), and the premotor and supplementary motor area (PMC&SMA) (22).

Participants completed the single-leg stance tasks to assess postural stability. Each participant performs two tasks: standing directly (stable surface) on the force platform and standing on a 5 cm thick foam pad (unstable surface) placed on the force platform on the affected limb, with their hands on the iliac crests, gaze fixed forward. The unaffected leg was flexing 45 degrees of knee flexion and 30 degrees of hip flexion. The order of testing conditions was randomized to minimize learning effects. Each trial began with a 60 s seated rest period to establish the baseline, followed by a 30 s single-leg stance task. Participants were given only one practice trial prior to the formal measurement to minimize any practice or learning effects (23). Three successful trials for each balance test were averaged for analysis. The time interval for breaks was 1-min seated rest between trials to minimize the potential influence of fatigue on postural performance. The trial was discarded and repeated if participants (1) shifted or hopped on the supporting leg, (2) hands moved from hips, or (3) made contact with the force platform using the contralateral limbs.

**Figure 1:**
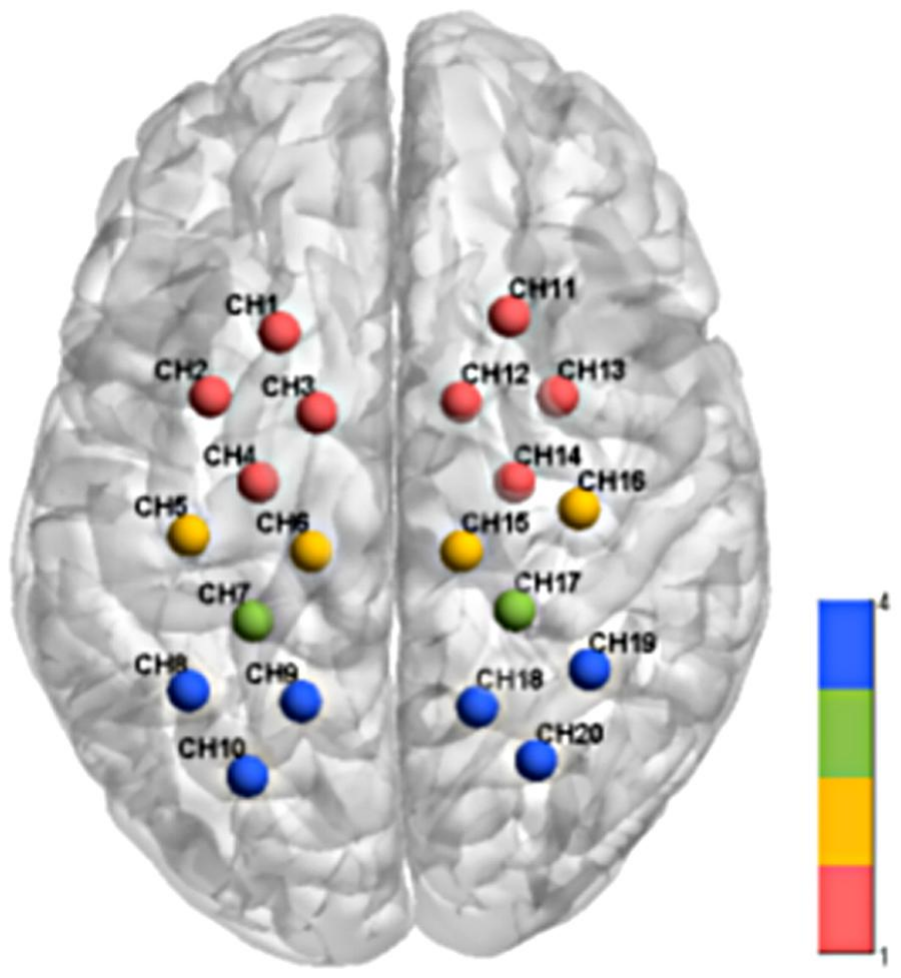
Schematic diagram of channel coordinates and corresponding brain regions of functional near-infrared brain imaging system. Different colors in the figure represent different brain region channels: the red channel is PMC&SMA; the yellow channel is M1; the green channel is S1; the blue channel is SAC.

Following data collection, a 3D digitizer (FASTRAK, Polhemus, Vermont) was used to determine the positions of the optodes. Channel coordinates were then registered to the Montreal Neurological Institute (MNI) space using the NIRS_SPM spatial probability registration toolkit (24), and the corresponding brain regions was identified (Figure 1).

### 2.3 Data reduction

#### 2.3.1 Center of pressure

The COP data were low-pass filtered with a Butterworth filter (cut-off frequency of 12 Hz) (9). The parameters were calculated based on the mathematical formula as follows (25):

$$COP\_RMS\_AP=\sqrt{\frac{\sum {\left(x_{i}-x\right)}^{2}}{N-1}}$$ (1)

$$COP\_RMS\_ML=\sqrt{\frac{\sum {\left(y_{i}-y\right)}^{2}}{N-1}}$$ (2)

where $x_{i}$ and $y_{i}$ represent of COP coordinates in anteroposterior (AP) and mediolateral (ML) directions, while *x* and *y* represent the average position of the COP in the AP and ML directions, and N represent number of points of the signal.

$$TOTEX=\underset{n}{\sum }\sqrt{{\left(X_{n+1}-X_{n}\right)}^{2}+{\left(Y_{n+1}-Y_{n}\right)}^{2}}$$ (3)

COP_Vel_Total=TOTEX/T (4)

where TOTEX represent total excursion length of the COP path, while T represent total duration of the signal (47).

$$95\text{\%}\;Area=2\pi \times \frac{N-1}{N-2}\times F_{0.95,2,N-2}\times \sqrt{RMS\_ML^{2}\times RMS\_AP^{2}\times COV^{2}}$$ (5)

where 95%Area represent the 95% confidence ellipse area of the COP, while $F_{0.95,2,N-2}$ denote the 0.95-quantile of the Fisher distribution with 2 and *n* - 2 degrees of freedom, and COV represent the covariance (25).

The nonlinear index sample entropy (SampEn) was calculated for both AP (SampEn_AP) and ML (SampEn_ML) directions. Sample entropy reflects the dynamic adaptability of the posture control system to task and environmental changes. Lower sample entropy values generally indicate increased attentional demands and reduced automaticity in postural control. The calculation formulas are as follows (48):

Step 1. Select two consecutive point sequences of length m from a vector $X_{N}=\{x_{1},x_{2}\ldots ,x_{N}\}$.

$$X_{m}(i)=\{x_{i,\ldots ,}x_{i+m-1}\}\;\mathrm{and}\;x_{m}(j)=\{x_{\;j,\ldots ,}x_{i+m-1}\}\{i,j\in [1,N-m],i\neq j\}$$ (6)

Step 2. Calculate the maximum distance:

$$d[X_{m}(i),X_{m}(j)]=max[\left|x_{i+k},x_{\;j+k}\right|]$$ (7)

Then compared it with the tolerance *r* of the repetitive sequence count:

$$d[X_{m}(i),X_{m}(j)]\leq r(k\in [0,m-1],r\geq 0)$$ (8)

Step 3. For the sequence $X_{m}(i)$, its count is defined as $B_{i}^{m}(r)$.

$B_{i}^{m}(r)$ is the average amount of $B_{i}^{m}(r)$, for i∈[1,N−m].

$A^{m}(r)$ is the average of $B_{i}^{m+1}(r)$.

$$\begin{array}{rl} \;\mathrm{Step}\;\;4.\; & SampEn(m,r,N)=\underset{N-\infty }{\lim}\left\{-\ln\left[\frac{A^{m}(r)}{B^{m}(r)}\right]\right\}\\ & =-\ln\left[\frac{A^{m}(r)}{B^{m}(r)}\right]\\ & =-\ln\left[\frac{{\left(N-m-1\right)}^{-1}\sum_{i=1}^{N-m-1}B_{i}^{m+1}(r)}{{\left(N-m\right)}^{-1}\sum_{i=1}^{N-m}B_{i}^{m}(r)}\right] \end{array}$$ (9)

Here, the parameters m and *r* are fixed values, where m represents the length of the sequence during comparison, and r is the tolerance for accepting a match. In this study, the recommended parameter values are (26): m = 2 and r = 0.2 * standard deviation.

#### 2.3.2 Hemodynamics

HbO_2_ data were preprocessed using the Homer2 toolbox, which is based on MATLAB (R2013b, MathWorks Inc, Natick, United States). The raw data were first converted to optical density signals, after which cubic spline interpolation correction was applied to correct the data and interpolate artifact-affected segments (27). Most systemic hemodynamic components were removed using a band-pass (0.01–0.1 Hz) filter (28), including fluctuations associated with Mayer waves (0.1 Hz), heart rate (1.6–1.8 Hz) and respiration (0.2–0.3 Hz) (22, 29). A differential path length factor (DPF) of 6 was used to account for the effective optical path between the source and detector. The optical density data were then transformed into changes in oxygenated (ΔHbO₂) and deoxyhemoglobin (HbR) concentrations using the modified Beer-Lambert law (30).The mean ΔHbO₂ value within the last 5 s of each sitting was selected as a baseline for correction, which was used to compute changes in oxyhemoglobin concentration (ΔHbO₂) (31, 32).

For each condition, fNIRS time series were segmented from 5 to 30 s after stimulus onset for the three valid trials. These segments were temporally aligned and averaged to emphasize task-related hemodynamic responses while reducing non-task-related fluctuations. Using the modified Beer-Lambert law, optical density was converted to blood oxygen concentration, including oxyhemoglobin concentration (HbO₂), deoxyhemoglobin concentration (HHb), and total hemoglobin concentration (HbT). Consistent with prior fNIRS research, brain activation was indexed using the integral of the HbO₂ hemodynamic response function (HRF) (33). Spatial patterns of cortical activation were visualized using the BrainNet Viewer toolbox in MATLAB.

### 2.4 Statistical analysis

All data were analyzed in SPSS 26.0 (IBMS, NY, USA) and presented as mean ± SD. The Shapiro–Wilk test was used to assess data normality. Group differences in baseline demographic characteristics were analyzed using independent samples *t*-tests. Two-way mixed-design ANOVAs, including the between-group factor (CAI vs. non-CAI) and the within-group factor (stable vs. unstable), were conducted to examine the main effects of conditions and group, as well as their interaction. Tests of simple main effects were conducted for any significant interaction between groups and support surface conditions. Bonferroni's *post-hoc* tests were performed to identify significant differences for significant main effects. For the near-infrared spectroscopy data, *p*-values were adjusted using the Benjamini-Hochberg procedure to control the false discovery rate (FDR) (29, 34). Partial eta squared (*η*2 p) was reported as the effect size for main and interaction effects for ANOVA results. The thresholds for *η*2 p were: 0.01–0.06 for small, 0.06–0.14 for medium, and greater than 0.14 for large effect sizes. The level of significance was set at *P* < 0.05.

## 3 Results

### 3.1 COP parameters

As shown in Table 2, significant group-by-condition interactions were detected in 95% Area (*P* = 0.023, *η*2 p = 0.152), RMS_ML (*P* = 0.004, *η*2 p = 0.228) and SampEn_ML (*P* = 0.015, *η*2 p = 0.172). *post-hoc* tests indicated that 95% Area was greater in the CAI group than in the non-CAI group on both the stable surface (*P* < 0.001) and the unstable surface (*P* = 0.035). Within the non-CAI group, the 95% Area was also higher on the unstable surface than on the stable surface (*P* = 0.001). For RMS_ML, the non-CAI group showed higher values on the unstable surface than on the stable surface (*P* < 0.001), and the CAI group exhibited greater RMS_ML than the non-CAI group on both the stable (*P* < 0.001) and unstable surfaces (*P* = 0.017). Additionally, SampEn_ML was lower in the CAI group than in the non-CAI group on the stable surface (*P* < 0.001), whereas SampEn_ML was higher on the unstable surface than on the stable surface (*P* < 0.001). Significant surface condition effects were found in both the CAI and non-CAI groups for COP_Vel_Total (*P* < 0.001, *η*2 p = 0.600) and RMS_AP (*P* < 0.001, *η*2 p = 0.344), as the unstable condition exhibited greater COP_Vel_Total and RMS_AP than the stable condition. And significant group effect was found in RMS_AP (*P* < 0.001, *η*2 p = 0.341), as the CAI group exhibited greater RMS_AP than the non-CAI group. For SampEn_AP, no significant group-by-condition interaction was observed (*P* = 0.610, *η*2 p = 0.008). In addition, neither the main effect of surface condition (*P* = 0.058, *η*2 p = 0.108) nor the main effect of group (*P* = 0.083, *η*2 p = 0.091) reached statistical significance.

**Table 2:** Results of COP parameters during single-leg stance on different support surfaces.

Indicator	Surface condition	CAI group	Non-CAI group	Condition effect		Group effect		Interaction	
				*P*	*η*2 p	*P*	η2 p	*P*	η2 p
COP_Vel_Total/ (㎜/s)	Stable	25.80 ± 6.15	21.63 ± 3.54	<0.001	0.600	0.055	0.110	0.264	0.039
	Unstable	29.48 ± 5.85	26.76 ± 5.55						
95% Area/ (㎜²)	Stable	1,459.04 ± 670.90^a^	789.27 ± 193.88	–	–	–	–	0.023	0.152
	Unstable	1,494.33 ± 325.17^a^	1,224.15 ± 386.60^b^						
RMS_ML/ (㎜)	Stable	8.25 ± 1.40^a^	6.06 ± 1.00	–	–	–	–	0.004	0.228
	Unstable	8.61 ± 0.86^a^	7.75 ± 1.63^b^						
RMS_AP/ (㎜)	Stable	10.37 ± 2.57	7.90 ± 1.23	<0.001	0.344	<0.001	0.341	0.348	0.028
	Unstable	11.32 ± 1.27	9.43 ± 1.78						
SampEn_ML	Stable	7.27 ±1.11^a^	8.75 ± 1.11	–	–	–	–	0.015	0.172
	Unstable	8.87 ± 1.79^b^	8.89 ± 1.63						
SampEn_AP	Stable	5.83 ± 1.46	6.50 ± 1.64	0.058	0.108	0.083	0.091	0.610	0.008
	Unstable	5.35 ± 1.06	6.21 ± 1.27						

COP_Vel_Total, Total velocity of the center of pressure (COP); 95% Area, The 95% confidence ellipse area of the COP trajectory; RMS_ML, Root mean square of COP displacement in the mediolateral (ML) direction; RMS_AP, Root mean square of COP displacement in the anteroposterior (AP) direction; SampEn_ML, Sample entropy of the COP signal in the mediolateral (ML) direction; SampEn_AP, Sample entropy of the COP signal in the anteroposterior (AP) direction. Surface condition, the type of support surface used in the test (stable and unstable), representing different standing surface conditions; CAI group, The group of participants with chronic ankle instability. Non-CAI group, The group of participants without chronic ankle instability.

^a^ Indicates significant difference compared with the non-CAI group.

^b^ Indicates significant difference compared with the stable surface condition, *P* < 0.05.

### 3.2 Cortical activation parameters

**Figure 2:**
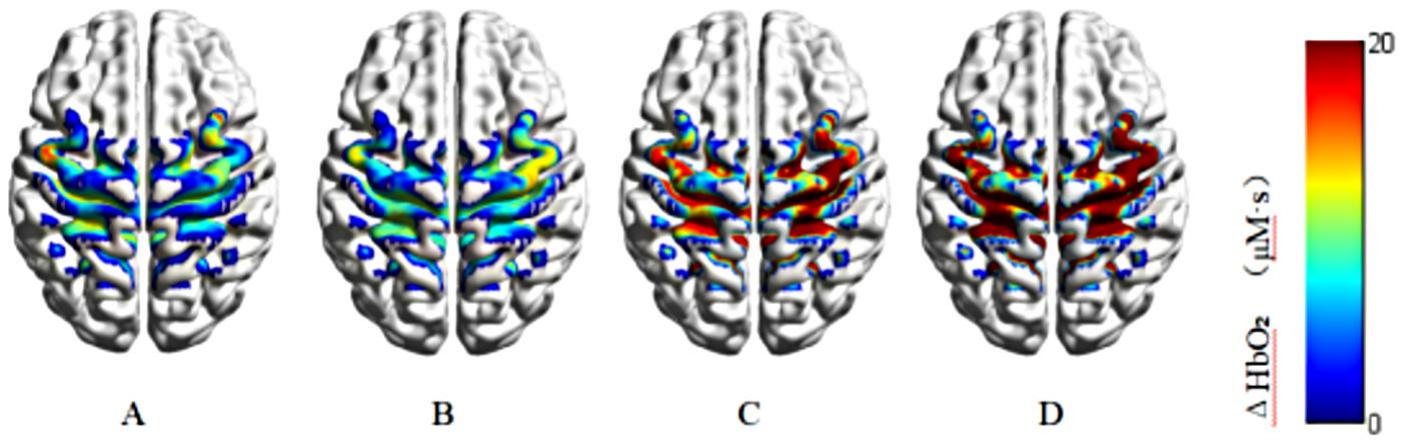
Comparison of cerebral cortex activation level between two groups of people when performing single-leg standing task under different support surfaces. The warmer the color (red), the greater the concentration of ΔHbO₂ and the higher the level of brain activation; the colder the color (blue), the smaller the change in the concentration of ΔHbO₂ and the lower the level of brain activation. **(A)** Non-CAI group—stable surface; **(B)** non-CAI group—unstable surface; **(C)** CAI group—stable surface; **(D)** CAI group—unstable surface.

As shown in Table 3, significant group-by-condition interactions were detected in channels 7 (*P* = 0.020, *η*2 p = 0.254),10 (*P* = 0.030, *η*2 p = 0.220), 11 (*P* < 0.001, *η*2 p = 0.522) and 13 (*P* = 0.030, *η*2 p = 0.212). Figure 2 illustrates the between-group comparisons of ΔHbO₂ under stable and unstable surface conditions at channels 7, 10, 11, and 13. *post hoc* tests indicated that ΔHbO₂ in the CAI group was higher on the stable surface than in the non-CAI group at channels 10 (*P* = 0.016) and 13 (*P* = 0.003). Additionally, the CAI group showed higher ΔHbO₂ on unstable surface than the non-CAI group at channels 7 (*P* < 0.001), 10 (*P* < 0.001), 11 (*P* < 0.001) and 13 (*P* < 0.001). Within the CAI group, ΔHbO₂ on the unstable surface was also higher than on the stable surface at these channels (channel 7: *P* = 0.003, channel 10: *P* = 0.009, channel 11: *P* = 0.003, channel 13: *P* = 0.003). Significant surface condition effects were found in channels 6 (*P* = 0.005, *η*2 p = 0.464), 9 (*P* = 0.030, *η*2 p = 0.189), and 15 (*P* = 0.005, *η*2 p = 0.380), as the unstable condition exhibited greater ΔHbO₂ at these channels than the stable condition. And significant group main effects were observed for all channels, as the CAI group exhibited greater ΔHbO₂ than the non-CAI group at all channels.

**Table 3:** Results of ΔHbO₂ during single-leg stance on different support surfaces (μM·s).

Brain area	Ch	CAI group		Non-CAI group		Condition effect		Group effect		Interaction		
		Stable	Unstable	Stable	Unstable	*P*	η2 p	*P*	η2 p	F	*P*	η2 p
Premotor and supplementary motor area (PMC&SMA)	1	18.83 ± 16.48	23.19 ± 13.38	4.75 ± 12.80	5.15 ± 6.62	0.289	0.035	<0.001	0.358	0.804	0.628	0.025
	2	19.06 ± 11.92	25.94 ± 7.52	17.74 ± 7.94	14.26 ± 6.72	0.405	0.022	0.006	0.210	6.607	0.050	0.171
	3	14.55 ± 7.21	14.01 ± 10.30	8.9 ± 13.94	7.29 ± 8.61	0.614	0.008	0.037	0.129	0.064	0.886	0.002
	4	23.19 ± 17.63	30.79 ± 6.64	7.04 ± 10.30	6.69 ± 9.78	0.166	0.059	<0.001	0.563	2.418	0.289	0.070
	11	23.65 ± 15.00^b^	44.51 ± 11.24^a^	19.05 ± 7.29	13.49 ± 6.18	–	–	–	–	34.981	<0.001	0.522
	12	30.19 ± 16.52	26.10 ± 4.10	12.93 ± 7.41	11.03 ± 7.23	0.229	0.045	<0.001	0.589	0.201	0.821	0.006
	13	17.17 ± 4.22^a^^b^	40.12 ± 14.28^a^^b^	5.93 ± 12.49	13.92 ± 8.98	–	–	–	–	8.591	0.030	0.212
	14	26.91 ± 19.22	32.67 ± 13.06	11.45 ± 11.31	16.09 ± 11.57	0.129	0.070	<0.001	0.392	0.028	0.886	0.001
Primary motor cortex (M1)	5	16.79 ± 8.39	16.41 ± 9.65	5.02 ± 10.32	6.38 ± 3.83	0.761	0.003	<0.001	0.391	0.296	0.787	0.009
	6	15.62 ± 10.19	28.58 ± 10.60	7.03 ± 6.49	12.15 ± 9.91	<0.001	0.464	<0.001	0.393	5.215	0.078	0.140
	15	24.49 ± 17.62	40.42 ± 6.40	4.07 ± 4.49	9.24 ± 7.46	<0.001	0.380	<0.001	0.751	5.092	0.078	0.137
	16	17.72 ± 16.35	22.91 ± 9.03	2.49 ± 3.58	6.19 ± 6.61	0.071	0.098	<0.001	0.560	0.099	0.886	0.003
Primary sensory cortex (S1)	7	9.23 ± 8.89^b^	22.66 ± 6.80^a^	8.05 ± 9.18	10.64 ± 8.45	–	–	–	–	10.875	0.020	0.254
	17	17.59 ± 11.94	14.82 ± 7.26	3.39 ± 9.58	−;0.03 ± 8.29	0.185	0.054	<0.001	0.557	0.021	0.886	0.001
Somatosensory association cortex (SAC)	8	18.87 ± 10.24	23.11 ± 11.26	10.52 ± 10.71	11.27 ± 9.59	0.298	0.034	0.001	0.303	0.550	0.687	0.017
	9	18.16 ± 12.12	29.03 ± 7.96	13.79 ± 10.50	9.72 ± 8.48	0.010	0.189	<0.001	0.536	6.699	0.050	0.173
	10	21.75 ± 17.19^a^^b^	32.56 ± 11.00^a^	9.67 ± 6.88	6.16 ± 9.03	–	–	–	–	9.017	0.030	0.220
	18	19.31 ± 14.08	22.32 ± 12.01	8.80 ± 6.01	5.34 ± 6.36	0.921	0.001	<0.001	0.449	2.060	0.322	0.060
	19	33.76 ± 16.16	31.64 ± 9.72	18.24 ± 8.82	22.15 ± 11.50	0.123	0.073	<0.001	0.463	0.509	0.687	0.016
	20	18.71 ± 17.36	19.65 ± 5.49	1.57 ± 12.44	8.87 ± 8.78	0.168	0.059	<0.001	0.432	1.182	0.518	0.036

Surface condition, the type of support surface used in the test (stable and unstable), representing different standing surface conditions; CAI group, The group of participants with chronic ankle instability. Non-CAI group, The group of participants without chronic ankle instability.

^a^ Significant difference compared with the non-CAI group.

^b^ Indicates significant difference compared with the stable surface condition, *P* < 0.05.

## 4 Discussion

This study compared the postural stability and sensorimotor cortex activation between individuals with and without CAI during single-leg stance on different surfaces. Compared with the stable surface, the unstable surface resulted in higher COP_Vel_Total and RMS_AP during single-leg stance. Other postural stability measures, including RMS_ML, 95% Area, and SampEn_AP, did not show consistent differences between surface conditions across both groups. In addition, increases in ΔHbO₂ were observed in the sensorimotor cortex during the unstable condition. These findings partially supported Hypothesis 1. Compared with the non-CAI group, the CAI group demonstrated lower SampEn_ML and higher COP_Vel_Total and RMS_AP, indicating reduced postural stability. Differences in 95% Area and RMS_ML between groups were more evident during the unstable surface condition. SampEn_AP did not differ significantly between groups. In addition, the CAI group showed greater ΔHbO₂ in multiple sensorimotor cortical regions, with between-group differences being more apparent during the unstable task. Compared with the non-CAI group, the CAI group demonstrated lower SampEn_ML and SampEn_AP, as well as higher 95% Area, RMS_ML, RMS_AP, and ΔHbO₂ across both surface conditions, with these differences being more pronounced on the unstable surface. These findings partially supported Hypothesis 2.

The study showed that individuals with CAI exhibited significantly poorer postural control than those without CAI, a difference that was more pronounced on the unstable surface. Impaired postural control is recognized as a critical factor in the progression from acute ankle sprain to CAI. Consistent with previous research, the CAI group showed larger RMS and 95% Area values under both surface conditions and a higher COP_Vel_Total on the unstable surface, which indicated impaired postural control (18). Recurrent sprains may lead to neuromuscular deficits that hinder the ability of individuals with CAI to maintain a single-leg stance, resulting in greater body sway than in healthy controls, particularly on unstable surfaces where these deficits may be further amplified, leading to poorer postural control performance manifested as larger postural oscillations. Notably, SampEn_ML was significantly higher in the CAI group on the unstable surface compared with the stable surface. Although higher SampEn is typically interpreted as reflecting increased system complexity and adaptability, lower SampEn is generally associated with a more rigid control strategy (35). In the present study, the elevated SampEn_ML on the unstable surface may reflect the heightened sensorimotor demands of instability. When attempting to compensate for proprioceptive deficits, individuals with CAI may experience impaired central information processing, which may reduce the accuracy of lower-limb spatial perception (36), and manifest in elevated SampEn alongside a compensatory strategy marked by larger and faster sway. Thus, the presence of sample entropy differences exclusively in the ML direction likely reflects a higher sensitivity of ML postural control to changes in neuromuscular coordination, while RMS captures amplitude-related changes in both directions. In summary, unstable surfaces impose greater demands on postural stability in individuals with CAI, yet their limited compensatory capacity is inadequate to overcome existing control deficits. Clarifying these differences may enhance understanding of the regulatory mechanisms underlying postural control in CAI.

The sensorimotor cortex plays a central role in postural control, and its activation varies with task difficulty (37), with increased task demands typically accompanied by increased activation of the PMC and SMA (38). In the present study, the CAI group exhibited significantly greater activation of S1 and PMC/SMA than the non-CAI group under both surface conditions, and displayed an additional increase in SAC activation on the unstable surface. According to the compensatory neural circuit utilization hypothesis (24), when primary sensorimotor pathways are compromised, the central nervous system may engage secondary or cross-regional circuits to maintain or restore motor function. Anatomical evidence further suggests that, compared with M1, the SMA possesses denser interhemispheric connections, making it is a key cortical hub for mediating neural interactions between the limbs (39). Previous research also indicated that S1 activation during balance control is substantially lower in individuals with CAI than in copers (13), implying that increased S1 activation may contribute to better functional recovery in copers and that unstable surfaces may offer a potential strategy for central modulation in individuals with CAI. Although no significant interaction between surface condition and group was observed for M1 activation in this study, M1 tended to exhibit increased activation on the unstable surface. This pattern likely reflects the mechanisms of motor regulation and sensory integration during postural control, in which sensory inputs are continuously reweighted according to task demands (40), and aligns with the view that M1, as a key node of the direct motor pathway, is more strongly engaged during automatized motor tasks. Overall, these findings suggest that individuals with CAI exhibit enhanced sensorimotor cortical activation during postural control, potentially reflecting a compensatory adaptation of the central nervous system.

Our findings indicate that individuals with CAI differ from those without CAI in the neural mechanisms supporting postural control. This observation has important implications for the design of brain-targeted interventions aimed at mitigating postural deficits. Previous studies suggest that anodal transcranial direct current stimulation may improve postural control by modulating cortical excitability (41); however, the variability of its effects across cortical regions underscores the need for more precise, region-specific modulation strategies in rehabilitation (42). In the present study, we found that participants with CAI showed distinct patterns of cortical activation and postural performance across different surface conditions in primary sensory and motor areas, the superior parietal region, and the PMC&SMA. Although brain-behavior relationships were not directly examined, the concurrent alterations in cortical activation and postural control identify these areas as potential neural substrates of postural control impairment in CAI and highlight them as promising targets for future therapeutic approaches (43). Integrating cortical-targeted interventions with neuromuscular training may therefore offer an effective strategy for enhancing postural stability in individuals with CAI.

Our findings observed that individuals with CAI place greater demands on cortical resources for motor control and sensory feedback to maintain balance. These finding provide new insights into the development of rehabilitation and assessment strategies for CAI. First, they underscore the importance of balance training under unstable surface conditions, as maintaining postural stability during more challenging tasks may require greater neural engagement (44), thereby promoting sensorimotor integration and neural adaptation. Accordingly, we propose incorporating unstable support surfaces into both balance assessments and rehabilitation programs for individuals with CAI. Second, prior research suggests that non-invasive brain stimulation represents a promising treatment strategy (45). By accentuating postural control deficits under unstable conditions, the present study identifies region-specific neural characteristics associated with balance control, thereby offering a theoretical framework for future non-invasive brain stimulation interventions targeting these regions. Third, our results indicate that balance assessments conducted across different support surface conditions, particularly those involving high-difficulty tasks such as landing onto an unstable surface, may more effectively detect postural control deficits in individuals with CAI.

This study has several limitations that should be considered when interpreting the findings. The sample consisted solely of university students aged 18–25 years, limiting the generalizability of the results to broader populations with CAI, including older adults, adolescents, and individuals with diverse occupational or activity backgrounds (46). In addition, we focused exclusively on brain regions associated with sensation and motor processing and did not assess whole-brain activity; therefore, we cannot fully characterize differences in global neural function between individuals with CAI and healthy controls during single-leg stance. Finally, the cross-sectional design precludes causal inference. Although individuals with CAI demonstrated greater brain activity during unstable surface stance, it remains uncertain whether this pattern reflects beneficial adaptation, a compensatory mechanism, or a modifiable response to training. Future longitudinal intervention studies are needed to determine whether training on unstable surfaces can produce durable changes in brain function and enhance symptoms and functional performance in this population.

## 5 Conclusion

Individuals with CAI exhibit impaired postural stability, particularly on unstable surfaces, and showed increased activation in the primary motor cortex, primary sensory cortex, somatosensory association cortex, and the premotor and supplementary motor areas. These findings indicate a compensatory neural mechanism, underscoring the essential contribution of cortical processes to the maintenance of postural control in this population.

## Data Availability

The original contributions presented in the study are included in the article/Supplementary Material, further inquiries can be directed to the corresponding author.

## References

[1] Gribble PA Bleakley CM Caulfield BM Docherty CL Fourchet F Fong DT 2016 Consensus statement of the international ankle consortium: prevalence, impact and long-term consequences of lateral ankle sprains. Br J Sports Med. (2016) 50(24):1493–5. 10.1136/bjsports-2016-096188 27259750

[2] Forsyth L Donovan L Martin-Smith R Rowe PL. Prevalence and impact of chronic ankle instability in female sport: a cross-sectional study. BMC Sports Sci Med Rehabil. (2025) 17:183. 10.1186/s13102-025-01211-5 40629426 PMC12235940

[3] Hu X Feng T Li P Liao J Wang L. Bilateral sensorimotor impairments in individuals with unilateral chronic ankle instability: a systematic review and meta-analysis. Sports Med-Open. (2024) 10:33. 10.1186/s40798-024-00702-y 38589676 PMC11001848

[4] Zhu X Wei F Li S Zhang T Shen P Fong DT Toe-out landing reduces anterior talofibular ligament strain while maintains calcaneofibular ligament strain in people with chronic ankle instability. J Sport Health Sci. (2025) 14:101035. 10.1016/j.jshs.2025.101035 40021056 PMC12478106

[5] Rosen AB Yentes JM McGrath ML Maerlender AC Myers SA Mukherjee M. Alterations in cortical activation among individuals with chronic ankle instability during single-limb postural control. J Athl Training. (2019) 54:718–26. 10.4085/1062-6050-448-17 PMC6602391 31162942

[6] Lin C-I Houtenbos S Lu Y-H Mayer F Wippert P-M. The epidemiology of chronic ankle instability with perceived ankle instability- a systematic review. J Foot Ankle Res. (2021) 14:41. 10.1186/s13047-021-00480-w 34049565 PMC8161930

[7] Xiao S Shen B Xu Z Zhan J Zhang C Han J Balance control deficits are associated with diminished ankle force sense, not position sense, in athletes with chronic ankle instability. Arch Phys Med Rehabil. (2024) 105(11):2127–34. 10.1016/j.apmr.2024.06.019 39009332

[8] Yu Y Liu DS Ruan B Gao Q. Advance in balance training for chronic ankle instability: a systematic review. Chin J Rehabil Theory Pract. (2019) 25(12):1374–83. 10.3969/j.issn.1006-9771.2019.12.003

[9] Ge Y Gao H Huang X Luo X Liu Y Wang D Effects of HD-tDCS combined with bosu ball training on static and dynamic postural stability among individuals with chronic ankle instability. Front Sports Active Living. (2025) 7:1618683. 10.3389/fspor.2025.1618683 PMC12239751 40636888

[10] Liu YH Dong SY Liu ZY Song P Shen PX. Unilateral chronic ankle instability affects bilateral postural stability, proprioception, plantar tactile sensation and muscle strength. CJTER. (2025) 29(17):3572–8. 10.12307/2025.663

[11] Maricot A Dick E Walravens A Pluym B Lathouwers E De Pauw K Brain neuroplasticity related to lateral ankle ligamentous injuries: a systematic review. Sports Med. (2023) 53(7):1423–43. 10.1007/s40279-023-01834-z 37155129

[12] Liu N Yang C Song Q Yang F Chen Y. Patients with chronic ankle instability exhibit increased sensorimotor cortex activation and correlation with poorer lateral balance control ability during single-leg stance: a FNIRS study. Front Hum Neurosci. (2024) 18:1366443. 10.3389/fnhum.2024.1366443 38736530 PMC11082417

[13] Ma T Xu X Li M Li Y Wang Y Li Q Cortical activation during single-legged stance in patients with chronic ankle instability. J Athl Train. (2023) 58:927–33. 10.4085/1062-6050-0363.22 36827609 PMC10784888

[14] Guo F Fu YM Li D Yuan WS Wang X. A study on modes of sensorimotor Cortex control of lower limb muscles under different support modes in national freestyle skiing aerial athletes. China Sport Science. (2021) 41(01):65–74+82. 10.16469/j.css.202101007

[15] Faul F Erdfelder E Lang AG Buchner A. G*power 3: a flexible statistical power analysis program for the social, behavioral, and biomedical sciences. Behav Res Methods. (2007) 39(2):175–91. 10.3758/BF03193146 17695343

[16] Gribble PA Delahunt E Bleakley C Caulfield B Docherty C Fourchet F Selection criteria for patients with chronic ankle instability in controlled research: a position statement of the international ankle consortium. Br J Sports Med. (2014) 48:1014–8. 10.1136/bjsports-2013-093175 24255768

[17] Li XK Feng R Rong K Sun XL Zhou ZP Yang C. Effects of ankle taping on knee and ankle biomechanics of individuals with chronic ankle instability inthe side-cutting and stop-jumping tasks. CJTER. (2026) 30(10):2422–9. 10.12307/2026.638

[18] Piri M Malmir K Otadi K Shadmehr A. Postural stability measures as diagnostic tools for chronic ankle instability: a comprehensive assessment. BMC Sports Sci Med Rehabil. (2025) 17:16. 10.1186/s13102-025-01064-y 39885584 PMC11784114

[19] McCamley J Bergamini E Grimpampi E. Balance on different unstable supports: a complementary approach based on linear and non-linear analyses. Med Biolog Eng Comput. (2022) 60(3):863–73. 10.1007/s11517-022-02504-4 35141819

[20] Xu G Zhou M Wang J Mao D Sun W. The effect of sensory manipulation on the static balance control and prefrontal cortex activation in older adults with mild cognitive impairment: a functional near-infrared spectroscopy (fNIRS) study. BMC Geriatr. (2024) 24:1020. 10.1186/s12877-024-05624-8 39702053 PMC11660590

[21] Seeck M Koessler L Bast T Leijten F Michel C Baumgartner C The standardized EEG electrode array of the IFCN. Clin Neurophysiol. (2017) 128:2070–7. 10.1016/j.clinph.2017.06.254 28778476

[22] Dong ZW Yu C Chen Y Ding JJ. Central nervous mechanisms underlying effects of cognitive impairment on dual-task stance:functional near-infrared spectroscopy analysis. CJTER. (2025) 29(17):3579–87. 10.12307/2025.632

[23] Zaghlul N Goh SL Razman R Danaee M Chan CK. Test-retest reliability of the single leg stance on a lafayette stability platform. PLoS One. (2023) 18:e0280361. 10.1371/journal.pone.0280361 36649257 PMC9844846

[24] Chen Y Cao Z Mao M Sun W Song Q Mao D. Increased cortical activation and enhanced functional connectivity in the prefrontal cortex ensure dynamic postural balance during dual-task obstacle negotiation in the older adults: a fNIRS study. Brain Cogn. (2022) 163:105904. 10.1016/j.bandc.2022.105904 36063567

[25] Quijoux F Nicolaï A Chairi I Bargiotas I Ricard D Yelnik A A review of center of pressure (COP) variables to quantify standing balance in elderly people: algorithms and open-access code. Physiol Rep. (2021) 9(22):e15067. 10.14814/phy2.15067 34826208 PMC8623280

[26] Kędziorek J Błażkiewicz M. Nonlinear measures to evaluate upright postural stability: a systematic review. Entropy. (2020) 22:1357. 10.3390/e22121357 33266239 PMC7760950

[27] Fishburn FA Ludlum RS Vaidya CJ Medvedev AV. Temporal derivative distribution repair (TDDR): a motion correction method for fNIRS. Neuroimage. (2019) 184:171–9. 10.1016/j.neuroimage.2018.09.025 30217544 PMC6230489

[28] Pinti P Scholkmann F Hamilton A Burgess P Tachtsidis I. Current Status and issues regarding Pre-processing of fNIRS neuroimaging data: an investigation of diverse signal filtering methods within a general linear model framework. Front Hum Neurosci. (2018) 12:505. 10.3389/fnhum.2018.00505 30687038 PMC6336925

[29] Li Y Xu Z Xie H Fu R Lo WLA Cheng X Changes in cortical activation during upright stance in individuals with chronic low back pain: an fNIRS study. Front Hum Neurosci. (2023) 17:1085831. 10.3389/fnhum.2023.1085831 36816497 PMC9936824

[30] Baker WB Parthasarathy AB Busch DR Mesquita RC Greenberg JH Yodh AG. Modified beer-Lambert law for blood flow. Biomed Opt Express. (2014) 5:4053–75. 10.1364/BOE.5.004053 25426330 PMC4242038

[31] Maidan I Nieuwhof F Bernad-Elazari H Reelick MF Bloem BR Giladi N The role of the frontal lobe in complex walking among patients with Parkinson’s disease and healthy older adults: an fNIRS study. Neurorehabil Neural Repair. (2016) 30:963–71. 10.1177/1545968316650426 27221042

[32] Dong Y Yang C Chen Y Pan F Wang J Zhang C. How aging impacts cortical dynamics and gait during dual-task turning revealed by fNIRS. Geroscience. (2025). 10.1007/s11357-025-01687-6 PMC12972402 40410646

[33] Yaramothu C Li X Morales C Alvarez TL. Reliability of frontal eye fields activation and very low-frequency oscillations observed during vergence eye movements: an fNIRS study. Sci Rep. (2020) 10:712. 10.1038/s41598-020-57597-4 31959829 PMC6971237

[34] Baldassarre A Lewis CM Committeri G Snyder AZ Romani GL Corbetta M. Individual variability in functional connectivity predicts performance of a perceptual task. Proc Natl Acad Sci USA. (2012) 109:3516–21. 10.1073/pnas.1113148109 22315406 PMC3295318

[35] Pettinato F Valle MS Cioni M Cirnigliaro L Rizzo R Barone R Dynamical complexity of postural control system in autism spectrum disorder: a feasibility study of linear and non-linear measures in posturographic analysis of upright posture. J NeuroEng Rehabil. (2024) 21:225. 10.1186/s12984-024-01520-9 39710690 PMC11664929

[36] Su YY Peng L Li W Li KQ Zhang Y Li J. Research progress of integrative NeuromuscularTraining for the rehabilitation and prevention ofChronic ankle instability. Chin Sport Sci Technol. (2023) 59(07):47–52. 10.16470/j.csst.2023014

[37] Boebinger S Payne A Martino G Kerr K Mirdamadi J McKay JL Precise cortical contributions to sensorimotor feedback control during reactive balance. PLOS Comput Biol. (2024) 20:e1011562. 10.1371/journal.pcbi.1011562 38630803 PMC11057980

[38] Li X Tang L Zhang Y Ye L Zhou L Tang M. The impact of interactive motor-cognitive dual tasking on brain activation, functional connectivity, and behavioral performance in healthy adults: an fNIRS study. Front Hum Neurosci. (2025) 19:1464617. 10.3389/fnhum.2025.1464617 40635721 PMC12237959

[39] Lee M Carroll TJ. Cross education: possible mechanisms for the contralateral effects of unilateral resistance training. Sports Med (Auckl NZ). (2007) 37:1–14. 10.2165/00007256-200737010-00001 17190532

[40] Cheng X Yang J Hao Z Li Y Fu R Zu Y The effects of proprioceptive weighting changes on posture control in patients with chronic low back pain: a cross-sectional study. Front Neurol. (2023) 14:1144900. 10.3389/fneur.2023.1144900 37273697 PMC10235490

[41] Xiao S Shen B Zhang C Xu Z Li J Fu W Effects of tDCS on foot biomechanics: a narrative review and clinical applications. Bioeng (Basel Switz). (2023) 10:1029. 10.3390/bioengineering10091029 PMC10525503 37760131

[42] Kim YK Shin SH. Comparison of effects of transcranial magnetic stimulation on primary motor cortex and supplementary motor area in motor skill learning (randomized, cross over study). Front Hum Neurosci. (2014) 8:937. 10.3389/fnhum.2014.00937 25477809 PMC4238326

[43] Zhang C Xiao S Shen B Xu Z Zhan J Li J Individualized transcranial direct current stimulation combined with foot core exercise improves foot and ankle sensorimotor function and static postural control in individuals with chronic ankle instability. J Neuroeng Rehabil. (2025) 22(1):182. 10.1186/s12984-025-01721-w 40841683 PMC12369118

[44] Legrand T Mongold SJ Muller L Naeije G Ghinst MV Bourguignon M. Cortical tracking of postural sways during standing balance (2024). Sci Rep. (2024) 14(1):30110. 10.1038/s41598-024-81865-2 39627308 PMC11615285

[45] Ma Y Yin K Zhuang W Zhang C Jiang Y Huang J Effects of combining high-definition transcranial direct current stimulation with short-foot exercise on chronic ankle instability: a pilot randomized and double-blinded study (2020). Brain Sci. (2020) 10(10):749. 10.3390/brainsci10100749 33080863 PMC7602979

[46] Miao Y Ge Y Wang D Mao D Song Q Wu R. Effects of visual disruption on static and dynamic postural control in people with and without chronic ankle instability. Front Bioeng Biotechnol. (2024) 12:1499684. 10.3389/fbioe.2024.1499684 39564099 PMC11574417

[47] Prieto TE Myklebust JB Hoffmann RG Lovett EG Myklebust BM. Measures of postural steadiness: differences between healthy young and elderly adults. IEEE Trans Biomed Eng. (1996) 43(9):956–66. 10.1109/10.532130 9214811

[48] Sun M Zhang F Lewis K Song Q Li L. The impact of Hoffmann reflex on standing postural control complexity in the elderly with impaired plantar sensation. Entropy (Basel). (2022) 25(1):64. 10.3390/e25010064 36673205 PMC9857425

